# Identification of prognostic metabolic genes in adrenocortical carcinoma and establishment of a prognostic nomogram: A bioinformatic study

**DOI:** 10.1097/MD.0000000000027864

**Published:** 2021-12-17

**Authors:** Qing Chen, Ziyu Ren, Dongfang Liu, Zongrui Jin, Xuan Wang, Rui Zhang, Qicong Liu, Wei Cheng

**Affiliations:** a Department of Endocrinology, the Second Affiliated Hospital of Chongqing Medical University, Chongqing, China; b Guangxi Medical University, Nanning, China.

**Keywords:** adrenocortical carcinoma, bioinformatics, metabolize, mRNA, prognosis, the cancer genome atlas

## Abstract

Adrenocortical carcinoma is an invasive malignancy with poor prognosis, high recurrence rate and limited therapeutic options. Therefore, it is necessary to establish an effective method to diagnose and evaluate the prognosis of patients, so as to realize individualized treatment and improve their survival rate.

This study investigated metabolic genes that may be potential therapeutic targets for Adrenocortical carcinoma (ACC). Level 3 gene expression data from the ACC cohort and the relevant clinical information were obtained from The Cancer Genome Atlas (TCGA) database. To verify, other ACC datasets (GSE76021, GSE19750) were downloaded from the Gene Expression Omnibus (GEO) database. The ACC datasets from TCGA and GEO were used to screen metabolic genes through the Molecular Signatures Database using gene set enrichment analysis. Then, the overlapping metabolic genes of the 2 datasets were identified.

A signature of five metabolic genes (CYP11B1, GSTM2, IRF9, RPL31, and UBE2C) was identified in patients with ACC. The signature could be used to divide the patients with ACC into high- and low-risk groups based on their median risk score. Multivariate Cox regression analysis was performed to determine the independent prognostic factors of ACC. Time-dependent receiver operating characteristic (ROC) curve analysis was conducted to assess the prediction accuracy of the prognostic signature. Last, a nomogram was established to assess the individualized prognosis prediction model.

The results indicated that the signature of 5 metabolic genes had excellent predictive value for ACC. These findings might help improve personalized treatment and medical decisions.

## 1 Introduction

Adrenocortical carcinoma (ACC) is a rare but aggressive endocrine malignancy with an incidence worldwide of 0.7 to 2.0 cases/million/year.^[1]^ It can occur at any age and has a bimodal incidence, predominantly affecting adults in their 40 to 50 second and children aged 1 to 5 years.^[2]^ ACC is a heterogeneous disease with multiple manifestations. For 40% to 60% of patients, the major presenting complaints are symptoms and signs of hormone excess.^[3,4]^ Roughly 10% to 15% of ACCs are incidentally diagnosed by imaging procedures for unrelated medical issues.^[5,6]^ Although our understanding of this disease has progressed tremendously over the last decade, the rate of early diagnosis of ACC is still low due to its rarity and lack of specific diagnostic criteria. Currently, surgical resection offers the only hope of a curative intervention in ACC. Initial staging is the most important factor in determining prognosis. However, even in patients with negative resection margin resection, recurrence and disease progression is still commonly seen, so that adjuvant therapy is frequently recommended. Currently, the only other adjuvant treatment shown to have effect is radiotherapy, however overall survival was not improved.^[7]^ Therefore, it is very important to find effective methods to improve the diagnosis and prognosis strategies of ACC.

Evidence suggests that the occurrence of tumors involves a variety of changes in cell metabolism. Fast-growing cancer cells produce adenosine triphosphate at a high rate of glycolysis, independent of the supply of oxygen, a process known as the Warburg effect.^[8]^ This change in metabolic pathways is a hallmark of cancer because it helps to absorb large amounts of nutrients into cellular building blocks, leading to an overproduction of the antioxidant glutathione, which leads to the creation of new cells.^[9]^ These changes lead to overexpression of metabolic genes. In most cases, metabolic changes are driven by oncogene-oriented metabolic reprogramming,^[10]^ which seems to be a common feature of highly invasive tumors, independent of carcinogenic origin.^[11]^ This change can be found in many tumors, such as pancreatic and colon cancers.^[12,13]^ However, there isn’t research shows the relationship between the metabolic genes and ACC. In this study, metabolic genes associated with the prognosis of ACC were identified on the basis of RNA-seq data from the cancer genome atlas (TCGA) through the Molecular Signatures Database (MSigDB),^[14]^ and a risk score model for ACC prognosis was constructed. A prognostic nomogram that combined prognostic gene trait risk models and clinical prognostic factors was established to predict overall survival (OS). The validity of the model was further verified through the geo database.

## 2 Materials and methods

### 2.1 Data source

The common shared data sets of 79 ACC patients and 128 normal tissues were downloaded from TCGA database (https://portal.gdc.cancer.gov/, accessed July 20, 2020) and GETx (Genotype-Tissue Expression database, https://www.gtexportal.org/home/index.html, accessed July 20, 2020). GTEX database is a high-throughput sequencing database covering many normal human tissues. Common data sources from 2 different data sets are standardized and combined. The clinical and pathologic data contain age, gender, tumor stage, TNM stage, survival time. All patient data involved in current study were acquired from the TCGA database and GETx database, and data retrieval and application complied with the TCGA and GETx publication guidelines and data access policies. Therefore, additional study approval by the local Ethical Review Committee was not required. other ACC datasets (GSE76021, GSE19750) were downloaded from the Gene Expression Omnibus (GEO) database. Additional ACC datasets (GSE76021, GSE19750) for validation were downloaded from the gene expression comprehensive database (GEO), which contained 29 ACC samples (GSE76021), 44 ACC samples (GSE19750) and 4 normal samples (GSE19750).

### 2.2 Metabolic gene screening

A list of metabolic genes was extracted from the datasets of Metabolic process (c2.cp.kegg.v7.0.symbols) from MSigDB (http://software.broadinstitute.org/gsea/msigdb/index.jsp).^[14]^ The expression data of these genes were screened from the ACC cases of TCGA and GEO. Metabolic prognostic genes were further evaluated with univariate Cox proportional hazard regression by using a “survival” package (version 3.2–3) on the R platform (version 3.6.3). Genes with *P* < .05 and |hazard ratio (HR)| > 1.00 were considered to be prognostic risk genes, and their expression levels were significantly associated with OS in ACC.

### 2.3 Signature development

The prognostic metabolic genes were analyzed using multivariate Cox regression analysis with OS as the dependent variable to evaluate their roles in predicting ACC survival. A prognostic risk score model was prepared via the linear combination of the expression levels of metabolic genes with the multivariate Cox regression coefficient (β) as the weight.^[15]^ The risk scores were calculated using the prognostic gene signatures. The risk score formula was as follows: risk score = expression of gene1 × β1 + expression of gene2 × β2 +⋯expression of gene n × βn.^[16–18]^ A total of 79 cases were divided into high- and low-risk groups based on the median risk score. |HR| > 1.0 and *P* < .05 were selected among the TCGA and GEO datasets as a cut-off. Then, 5 genes were selected for signature development. Receiver operating characteristic (ROC) curve refers to the receiver operating characteristic curve. It is a comprehensive index reflecting the continuous variables of sensitivity and specificity. It reveals the relationship between sensitivity and specificity by composition. A ROC curve was established over time on the R platform to assess the accuracy of the risk score model for predicting the prognosis of ACC.

### 2.4 Functional and pathway enrichment analyses

Gene ontology (GO) simply annotates the gene products from the aspects of function, involved biological pathways and localization in cells. Go enrichment analysis can be used to understand the biological functions, pathways or cell localization of gene enrichment. Kyoto Encyclopedia of Genes and Genomes (KEGG) is a comprehensive database integrating genome, chemistry and system function information. KEGG pathway stores the information of many gene pathways in different species. The functional enrichment analyses of the metabolic genes mainly involving GO terms and KEGG pathway analysis were carried out using the “cluster Profiler” R package.^[19]^ GO analysis revealed the functions of the metabolic genes in biological processes (BP), cellular components, and molecular functions, and the KEGG analysis showed the pathway enrichment of the metabolic genes. *P* < .05 was considered statistically significant.

### 2.5 Predictive nomogram construction and validation

Prognostic characteristics based on metabolic gene expression were analyzed to further evaluate the prognostic model. After collinearity was tested, a Norman diagram was predicted using a stepwise Cox regression model to predict the 1-, 3-, and 5-year OS rates of the patients with ACC the datasets from TCGA and GEO. Kaplan–Meier analysis and area under the curve comparison of the ROC curve were applied to predict and observe the OS rate for assessing the performance of the prognostic nomogram. We not only compared the clinical outcomes of the low risk and high risk groups, but also assessed the prognostic value of ACC using nomogram's risk score. The potential application of risk scores in the prediction of clinical status was also discussed.

### 2.6 Statistical analysis

Lasso (least absolute shrinkage and selection operator) method is a compression estimation method based on the idea of reducing variable set (order reduction). By constructing a penalty function, it can compress the coefficients of variables and make some regression coefficients become 0, so as to achieve the purpose of variable selection. Compared with ordinary logistic regression, a bias term is added to the regression optimization function to reduce the influence of collinearity and reduce the model variance. Therefore, it is used to screen important genes related to ACC. Kaplan–Meier survival analysis statistical method is a survival analysis method that can investigate single factors and hierarchical control confounding factors, which is suitable for large samples or small samples. Kaplan–Meier survival analysis by log-rank test was conducted to identify the metabolic genes associated with the prognosis of ACC. Univariate, multivariate, and Cox regression analyses were executed in R. Univariate and multivariate Cox regression analyses as much performed to assess survival, HRs and 95% confidence intervals were calculated to identify OS-associated genes. Statistical significance was set at *P* < .05.

## 3 Results

### 3.1 Identification of metabolic genes with prognostic value

**Figure 1:**
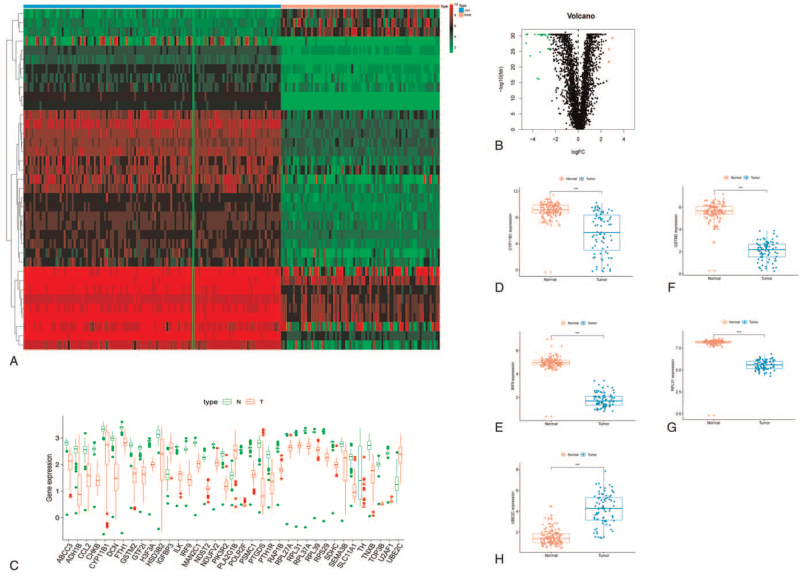
Expression of 37 metabolism related genes and 5 prognostic core genes in Adrenocortical carcinoma (ACC); (A) Expression Heatmap of all genes in normal and tumor tissues, with red representing upregulation and green representing downregulation. Data were obtained from TCGA, (B) Differential gene volcano map, red represents upregulation and green represents downregulation. (C) Expression of 37 differentially metabolized genes between normal tissues and tumors, red represents upregulation and green represents downregulation. (D) CYP11B1, (E) GSTM2, (F) IRF9, (G) RPL31, (H) UBE2C.

**Figure 2:**
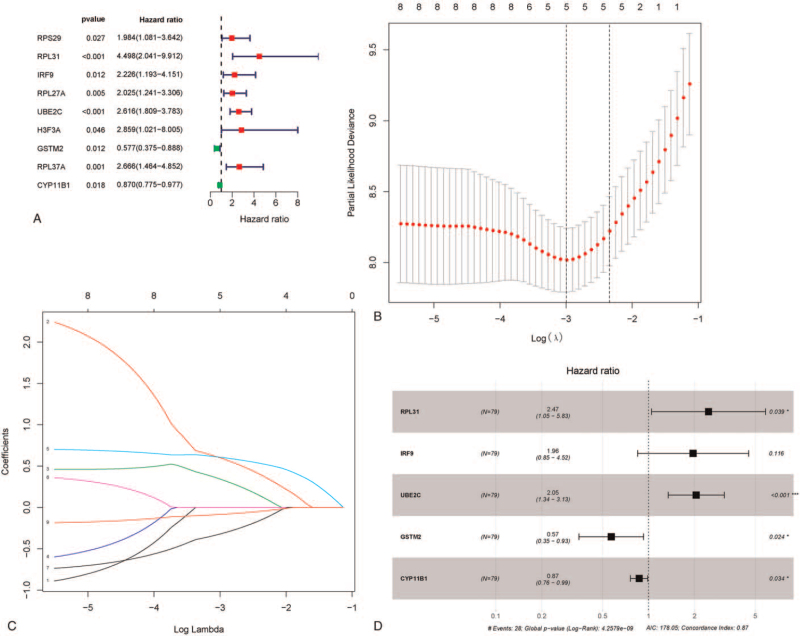
Screening of 5 core genes; (A) Results of univariate analysis based on differential metabolic gene expression, (B, C) The process of lasso regression analysis, (D) Multivariate analysis based on effective differential metabolic gene expression after univariate analysis.

Clinical information and gene expression profiles of 79 ACC cases and 128 normal tissues downloaded from the database of TCGA were used for further analysis. A total of 36521 metabolic genes were selected from MSigDB v4.0^[14]^ Metabolic process (c2.cp.kegg.v7.0.symbols). For validation, gene expression microarray datasets GSE76021 and GSE19750 were downloaded from the GEO dataset. We extracted 3527 metabolism related genes expression quantities from TCGA and GTEX, and then compared the expression quantities by volcano map of differential in tumor tissues and normal tissues (Fig. 1B), resulting in 37 differential genes (Fig. 1C), and taked heatmap of differentially expressed metabolic genes in the Gene Expression Omnibus cohort (Fig. 1A). Then, 5 best modeled prognostic metabolic related genes (11β-hydroxylase P450c11 [CYP11B1], glutathione-S-transferases mu 2 [GSTM2], interferon regulatory factor 9 [IRF9], ribosomal protein L31 [RPL31] and ubiquitin-conjugating enzyme E2C [UBE2C]) were identified by univariate analysis, multivariate analysis and lasso regression analysis, and were used to establish the metabolic gene signature (Fig. 1 DEFGH) and the results are shown in Figure 2, Tables 1 and 2.

**Table 1:** xxxx.

Id	HR	HR.95L	HR.95H	*P* value
RPS29	1.983898167	1.080620945	3.642213262	.0270979
RPL31	4.49757919	2.040765418	9.912074358	.000192074
IRF9	2.225511025	1.193282913	4.150649664	.011881593
RPL27A	2.025251643	1.240650243	3.306043938	.004766747
UBE2C	2.616123087	1.809031407	3.783295293	3.231040182
H3F3A	2.858683906	1.020821803	8.005387082	.045588031
GSTM2	0.577364214	0.37549014	0.887771475	.012340497
RPL27A	2.665524947	1.464434062	4.851719466	.001335247
GSTM2	0.870139614	0.775185453	0.976724918	.018303571

**Table 2:** xxxx.

Id	Coef	HR	HR.95L	HR.95H	*P* value
RPL31	0.904238627	2.470050577	1.047129791	5.826545957	.038909596
IRF9	0.671920087	1.957993232	1.047129791	4.521446836	.115586995
UBE2C	0.717969594	2.050266109	1.343038837	3.129910321	.000879668
GSTM2	−0.561156263	0.570548978	0.351050053	0.927292655	.023537575
CYP11B1	−0.141209604	0.868307292	0.761979656	0.989472026	.034110122

### 3.2 Prognostic model construction and receiver operating characteristic curve analysis

**Figure 3:**
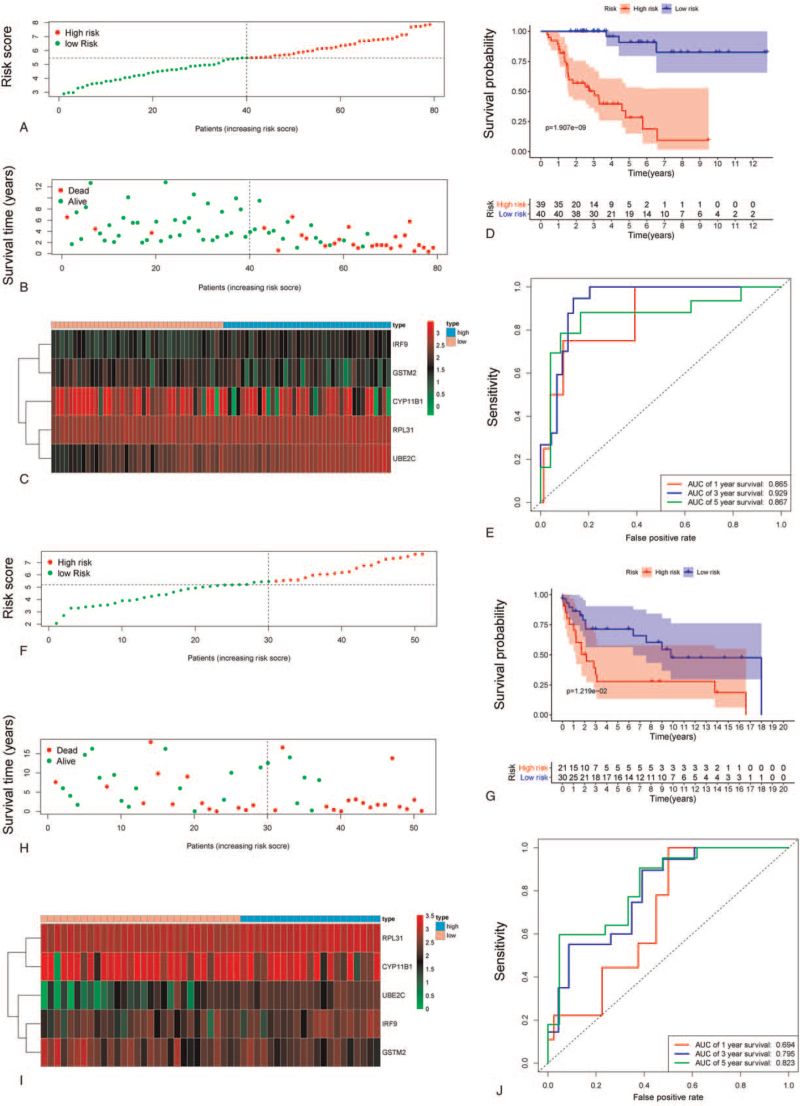
The performance of prognostic evaluation model in TCGA and geo database; (A, B) Death risk factor score based on TCGA database, (C) Heat map of 5 core genes based on TCGA database, (D) Survival curve of high and low risk groups in prognosis evaluation model based on TCGA database, (E) ROC curve of prognosis evaluation model based on TCGA database, (F,G) Death risk factor score based on GEO database, (H) Heat map of 5 core genes based on GEO database, (I) Survival curve of high and low risk groups in prognosis evaluation model based on GEO database, (J) ROC curve of prognosis evaluation model based on GEO database.

The signatures of 5 metabolic genes were developed using a risk-scoring approach. The 79 ACC patients in the database from TCGA and GETx were divided into high- and low-risk groups based on their median risk score. The Kaplan–Meier analysis in the GEO cohort are shown in Figure 3AB, and in the TCGA cohort are shown in Figure 3FH. Heatmap of differentially expressed metabolic genes in TCGA cohort (Fig. 3C) and Gene Expression Omnibus (GEO) cohort (Fig. 3I). Survival analysis showed that the OS of the patients in the high-risk group was shorter (median survival time of high risk and low risk: 748 vs 1547 day) than it was in the low-risk group (*P* < .001, Fig. 3D). Validation by the GEO database further confirmed that the survival rate of the high-risk score group was lower than that of the low-risk group (*P* < .001, Fig. 3G). Our results showed that the prognostic signature from the database from TCGA in the current study performed well in predicting 1-, 3-, and 5-year survival rates. The area under the curve values for 1-, 3-, and 5-year survival were 0.865, 0.929, and 0.867, respectively (Fig. 3E). The prognostic signatures of the GEO database also performed well in predicting 1-, 3-, and 5-year survival rates (Fig. 3J).

### 3.3 Predictive nomogram construction and validation

**Figure 4:**
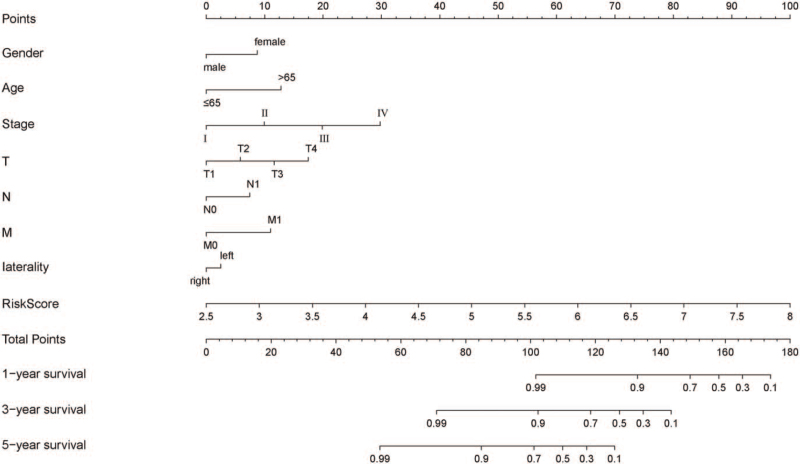
Nomogram of prognosis evaluation model composed of 5 core genes.

The nomogram calculates the predictive value for an outcome event for that individual by constructing a multivariate regression model, allowing visual representation of the model's influence on the outcome event. Nomogram survival analysis was used to investigate the relationship between OS risk score and clinical characteristics of ACC patients. A nomogram was drawn with RMS and its auxiliary packages based on the clinical information of ACC and the risk score. The results showed that the prognostic indicators of the risk score significantly influenced the risk points, whereas other clinical features had a lower effect on the risk points (Fig. 4). In our nomogram, the disadvantage was that the prognostic signature of tumor stage could not perform well in ACC.

### 3.4 Functional enrichment analysis of genes

**Figure 5:**
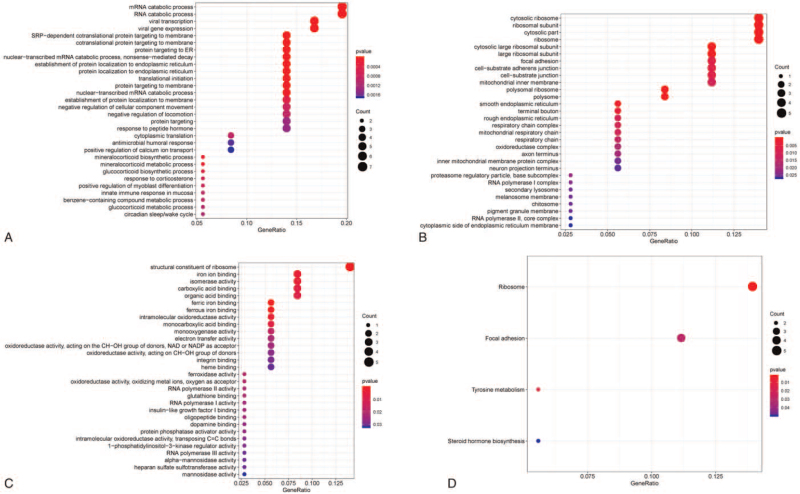
Functional Enrichment Analysis of Genes; (A) Biological process (BP), (B) Cellular component (CC), (C) Molecular function (MF), (D) Kyoto Encyclopedia of Genes and Genomes (KEGG).

**Figure 6:**
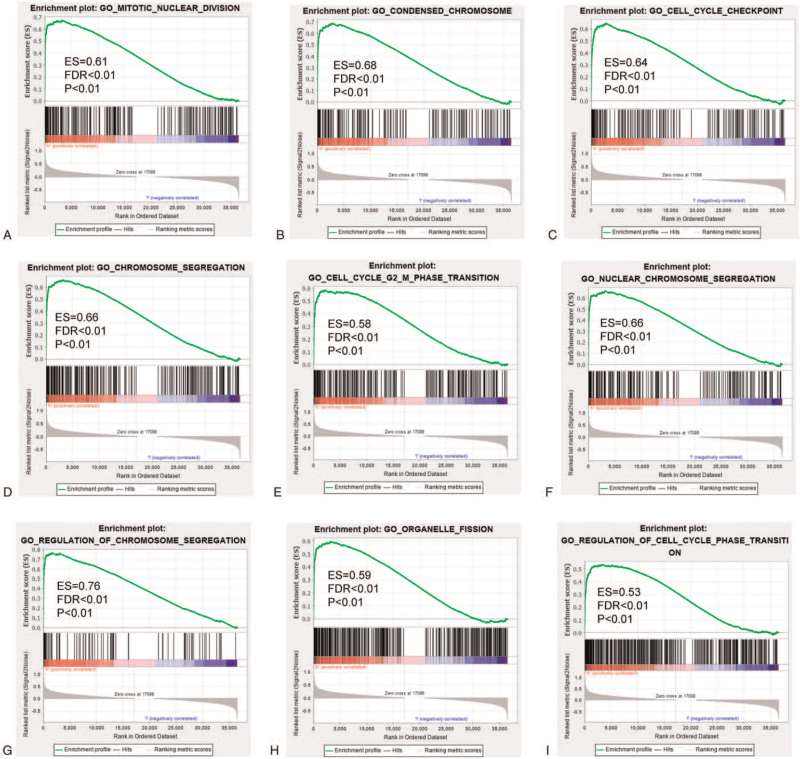
Gene set enrichment analysis (GSEA) showed that 5 genes were mainly associated with condensed chromosome (B), regulation of chromosome segregation(G), and cell cycle transition(E,I).

Metabolic genes were subjected to functional enrichment analysis by applying the cluster Profiler R package. The metabolic genes from the database from TCGA in the BP analysis were mainly enriched in nuclear-transcribed mRNA catabolic process and protein targeting to ER. The genes in the cellular component analysis were significantly enriched in cytosolic part and mitochondrial inner membrane. The genes in the molecular function analysis were mainly enriched in structural constituent of ribosome and intramolecular oxidoreductase activity (*P* < .05, Fig. 5A, B, C). KEGG analysis suggested that most of the metabolic gene pathways were significantly linked to ribosome and focal adhesion (*P* < .05, Figure 5D). Gene set enrichment analysis was used to clarify the main biological functions of 5 node genes. The results showed that they were mainly associated with condensed chromosome, regulation of chromosome segregation, and cell cycle transition (Fig. 6).

## 4 Discussion

Adrenal tumors are very common, and the majority are small benign nonfunctional adrenocortical adenomas (ACA).^[20]^ ACC, in contrast, is a rare cancer of the adrenal cortex. Despite its rarity, outcomes for patients diagnosed with ACC remain dismal, with a 5-year overall survival of ∼35%.^[21]^ Clinically apparent adrenocortical hormone production is evident in up to 45% to 70%.^[22]^ However, syndromes of hormone excess are often not readily recognized by physicians, leading to delay in diagnosis and subsequent surgical and/or medical therapy. Typically, the ideal treatment for ACC is surgical intervention, but ∼75% of all patients will ultimately develop metastases.^[23]^ Patients with metastatic disease mainly receive radiation therapy, chemotherapy, or immunotherapy. Mitotane is a unique antineoplastic agent used solely in the treatment of ACC, sometimes paired with palliative surgery. Several mechanisms have been postulated for its action including alteration of mitochondrial respiratory chain activity by inducing cytochrome c oxidase defect in adrenocortical cells. However, these treatments are often ineffective. These challenges call for an urgent need to find more effective methods to improve strategies for ACC diagnosis and prognosis.

Metabolic reprogramming is considered a hallmark of cancer because it is the most common physiological change in cancer cells.^[24]^ In recent years, several metabolic gene models have been established for various types of cancer, such as colon cancer,^[13]^ thyroid cancer,^[25]^ and ovarian cancer.^[26]^ Therefore, understanding changes in metabolite levels associated with ACC will help in the search for accurate and clinically useful biomarkers and therapeutic targets. Comprehensive analysis of metabolomics and transcriptomics data is an advanced method for finding reliable metabolic biomarkers. However, as far as we know, no metabolic gene signature has been set up for ACC. So, this study attempted to identify differentially expressed metabolic genes and establish a useful metabolic gene signature to predict clinical outcomes for patients with ACC.

In this study, we identified a total of 37 dysregulated metabolic genes in ACC using GEO and TCGA data. Then, 5 identified genes, CYP11B1, GSTM2, IRF9, RPL31, and UBE2C were found to be associated with a high risk in the databases from TCGA and GEO. The signature of 5 metabolic genes was developed using a risk score method, and the patients with ACC were divided into low- and high-risk groups based on their median risk score. Analysis of the gene set enrichment analysis indicated that these genes were mainly enriched in cell growth and division in the high-risk groups. And these pathways of active cell growth may have an important relationship with highly active tumor growth in the high-risk group. Multivariate Cox regression analysis was conducted to determine the independent prognostic factors of ACC. Then, the prognostic signature was comprehensively analyzed on the basis of metabolic gene expression. Kaplan–Meier analysis and the area under the curve comparison of the ROC curve confirmed that the 5 metabolic gene signatures were reliable for OS prediction. Next, a nomogram was established and integrated with a signature of 5 metabolic genes and clinical data, and OS was accurately predicted. Furthermore, a prognostic nomogram was established based on the risk score and 7 clinicopathological factors. The prognostic accuracy of the model was confirmed by the ROC curve and the corresponding calibration curve. These results indicate that the signature not only serves as a biomarker for ACC, independent of clinicopathological features, but also predicts clinical outcomes in ACC patients.

In our current study, 5 genes associated with prognosis of ACC were identified: CYP11B1, GSTM2, IRF9, RPL31, and UBE2C. Most of the 5 genes of the signature have been reported in various types of cancer. CYP11B1 is a Protein Coding gene which provides instructions for making an enzyme called 11-beta-hydroxylase. This enzyme is found in the adrenal glands, which are located on top of the kidneys. The 11-beta-hydroxylase enzyme is a member of the cytochrome P450 family of enzymes. These enzymes are involved in the formation and breakdown of various molecules within cells. The 11-beta-hydroxylase enzyme helps produce hormones called cortisol and corticosterone. Mutations in this gene cause congenital adrenal hyperplasia due to 11-beta-hydroxylase deficiency. Mitotane as an adjuvant therapy for ACC patients, it is metabolites can inhibit several enzymes in the adrenocortical steroidogenesis pathway, mainly at the level of the cholesterol side-chain cleavage enzymes CYP11A1 and CYP11B1.^[27,28]^ CYP11B1 is reliable biomarkers for the histopathological subtyping of functional benign adrenocortical tumors (ACT) and may offer some value in the histopathological diagnosis of malignant ACT.^[29]^ Compared with normal tissue, this gene is low expressed in ACC.

The GO annotations related to GSTM2 include *protein homodimerization activity and enzyme binding*. GSTM2 is a Protein Coding gene. This gene encodes a glutathione S-transferase that belongs to the mu class. The mu class of enzymes functions in the detoxification of electrophilic compounds, including carcinogens, therapeutic drugs, environmental toxins and products of oxidative stress, by conjugation with glutathione. Genetic variants of glutathione S-transferase as possible risk factors for carcinoma. The genetic variations can change an individual's susceptibility to carcinogens and toxins as well as affect the toxicity and efficacy of certain drugs.^[30,31]^ Diseases associated with GSTM2 include Barrett's Adenocarcinoma^[32]^ and Hepatocellular Carcinoma.^[33]^ Therefore, the low GSTM2 expression in ACC might be the cause of ACC pathogenesis and progression.

Diseases associated with IRF9 include Immunodeficiency 65 Viral Infections^[34]^ and Lymphocytic Choriomeningitis.^[35]^ Among its related pathways are Interferon Pathway and JAK-STAT signaling pathway. GO annotations related to this gene include *DNA-binding transcription factor activity.* Studies have shown that the triple negative breast cancer inherent interferon regulatory factor IRF9 as a marker of active intratumoral type I and II interferon signaling and reduced risk of distant relapse.^[36]^

RPL31 encodes a ribosomal protein that is a component of the 60S subunit. The protein belongs to the L31E family of ribosomal proteins. It is located in the cytoplasm. Higher levels of expression of this gene in familial adenomatous polyps compared to matched normal tissues have been observed. Diseases associated with RPL31 include Diamond-Blackfan Anemia^[37]^ and Combined Oxidative Phosphorylation Deficiency 1. Among its related pathways are rRNA processing in the nucleus and cytosol and Viral mRNA Translation. GO annotations related to this gene include *structural constituent of ribosome.*

UBE2C encodes a member of the E2 ubiquitin-conjugating enzyme family. The encoded protein is required for the destruction of mitotic cyclins and for cell cycle progression, and may be involved in cancer progression. Diseases associated with UBE2C include Breast cancer^[38]^ and Gastric Cancer.^[39]^ Among its related pathways are Gastric Cancer Network 2 and Regulation of activated PAK-2p34 by proteasome mediated degradation.

This study has some limitations. First, our clinical information was mainly obtained from databases from TCGA and GEO. Some patients’ clinical information was incomplete, and detailed data on patient prognosis were unavailable. Second, we were unable to evaluate the predictive accuracy of the nomogram by external validation. The expression level of prognosis-related genes and their molecular mechanisms in the pathogenesis and progression of ACC should be further explored experimentally. The screened genes could be verified through real-time PCR and Western blot.

## 5 Conclusion

We successfully established a novel metabolic gene signature in ACC. This signature was not only able to predict OS in ACC patients but was also used as a guide for the diagnosis and therapy of ACC.

## Author contributions

**Formal analysis:** Qing Chen, Zongrui Jin, Xuan Wang.

**Funding acquisition:** Dongfang Liu.

**Investigation:** Ziyu Ren.

**Methodology:** Qing Chen, Rui Zhang.

**Resources:** Qing Chen, Qicong Liu.

**Software:** Qing Chen.

**Supervision:** Dongfang Liu.

**Writing – original draft:** Qing Chen.

**Writing – review & editing:** Dongfang Liu, Wei Cheng.
